# Preventing muscle wasting: pro‐insulin C‐peptide prevents loss in muscle mass in streptozotocin‐diabetic rats

**DOI:** 10.1002/jcsm.13210

**Published:** 2023-03-06

**Authors:** Samantha Maurotti, Roberta Pujia, Angelo Galluccio, Saverio Nucera, Vincenzo Musolino, Rosario Mare, Miriam Frosina, Francesca Rita Noto, Vincenzo Mollace, Stefano Romeo, Arturo Pujia, Tiziana Montalcini

**Affiliations:** ^1^ Department of Clinical and Experimental Medicine University Magna Græcia Catanzaro Italy; ^2^ Department of Medical and Surgical Science University Magna Græcia Catanzaro Italy; ^3^ Department of Health Science University Magna Græcia Catanzaro Italy; ^4^ Department of Molecular and Clinical Medicine University of Gothenburg Gothenburg Sweden; ^5^ Research Center for the Prevention and Treatment of Metabolic Diseases (CR METDIS) University Magna Græcia Catanzaro Italy

**Keywords:** C‐peptide, muscle mass, type 1 diabetes, atrophy, muscle‐specific E3 ubiquitin ligase

## Abstract

**Background:**

C‐peptide therapy exerts several positive actions on nerves, vasculature, smooth muscle relaxation, kidney function and bone. To date, the role of C‐peptide in preventing type 1 diabetes‐related muscle atrophy has not been investigated. Our aim was to evaluate if C‐peptide infusion prevents muscle wasting in diabetic rats.

**Methods:**

Twenty‐three male Wistar rats were randomly divided into three groups: normal control group, diabetic group and diabetic group plus C‐peptide. Diabetes was induced by streptozotocin injection, and C‐peptide was administered subcutaneously for 6 weeks. The blood samples were obtained at baseline, before streptozotocin injection and at the end of the study to assess C‐peptide, ubiquitin and other laboratory parameters. We also tested the ability of C‐peptide to regulate the skeletal muscle mass, the ubiquitin–proteasome system, the autophagy pathway as well as to improve muscle quality.

**Results:**

C‐peptide administration reversed hyperglycaemia (*P* = 0.02) and hypertriglyceridaemia (*P* = 0.01) in diabetic plus C‐peptide rats compared with diabetic control rats. The diabetic‐control animals displayed a lower weight of the muscles in the lower limb considered individually than the control rats and the diabetic plus C‐peptide rats (*P* = 0.03; *P* = 0.03; *P* = 0.04; *P* = 0.004, respectively). The diabetic‐control rats presented a significantly higher serum concentration of ubiquitin compared with the diabetic plus C‐peptide and the control animals (*P* = 0.02 and *P* = 0.01). In muscles of the lower limb, the pAmpk expression was higher in the diabetic plus C‐peptide than the diabetic‐control rats (in the gastrocnemius, *P* = 0.002; in the tibialis anterior *P* = 0.005). The protein expression of Atrogin‐1 in gastrocnemius and tibialis was lower in the diabetic plus C‐peptide than in diabetic‐control rats (*P* = 0.02, *P* = 0.03). After 42 days, the cross‐sectional area in the gastrocnemius of the diabetic plus C‐peptide group had been reduced by 6.6% while the diabetic‐control rats had a 39.5% reduction compared with the control animals (*P* = 0.02). The cross‐sectional area of the tibialis and the extensor digitorum longus muscles was reduced, in the diabetic plus C‐peptide rats, by 10% and 11%, respectively, while the diabetic‐control group had a reduction of 65% and 45% compared with the control animals (both *P* < 0.0001). Similar results were obtained for the minimum Feret's diameter and perimeter.

**Conclusions:**

C‐peptide administration in rats could protect skeletal muscle mass from atrophy induced by type 1 diabetes mellitus. Our findings could suggest that targeting the ubiquitin–proteasome system, Ampk and muscle‐specific E3 ubiquitin ligases such as Atrogin‐1 and Traf6 may be an effective strategy for molecular and clinical intervention in the muscle wasting pathological process in T1DM.

## Introduction

It is well recognised that diabetes per se reduces muscle size and strength.^1^, ^2^, ^3^, ^4^, ^5^, ^6^ Individuals with newly diagnosed or poorly controlled type 1 diabetes mellitus (T1DM) are particularly affected by muscle atrophy.^6^, ^7^ However, there are currently no approved drugs to treat severe muscle loss in patients with diabetes.

Although several studies indicate that the insulin‐signalling pathway activates protein synthesis,^2^, ^8^, ^9^ administration of insulin has been reported to have no effect on skeletal muscle in humans.^10^ Rodent and in vitro models seem to suggest that insulin's role in inhibiting protein degradation in muscle is more important than in protein synthesis.^11^, ^12^ The decrease in insulin production causes accelerated muscle proteolysis via the ubiquitin–proteasome system (UPS).^13^ Transcripts of the UPS are more prevalent in muscle biopsies from individuals affected by T1DM who have not been treated with insulin.^14^ Studies on skeletal muscle atrophy have also highlighted that the metabolic sensor 5′ AMP‐activated protein kinase (AMPK) is a crucial regulator of muscle plasticity.^15^ AMPK coordinates anabolic and catabolic pathways.^15^ Mammalian FOXOs serve *specific* roles in controlling the activation in proteolytic and autophagy pathways that occur upon insulin deficiency.^14^, ^16^ FOXOs also upregulate muscle‐specific E3 ubiquitin ligases such as Atrogin‐1 and Murf‐1 (also known as Fbxo32 and Trim63, respectively).^14^, ^16^ These pathways may thus represent potential therapeutic targets to prevent muscle loss in patients with diabetes.

The burden placed on the healthcare system as a result of muscle loss and sarcopenia is expected to increase significantly in the future. In fact, because of muscle loss, patients with diabetes have difficulty in performing basic physical tasks such as standing for long period and walking long distances. The annual incidence of falls for patients with diabetes over the age of 65 is estimated to be 39%.^17^ Therefore, the development of preventive and therapeutic strategies against muscle loss in patients with diabetes is imperative.

A retrospective analysis of the Diabetes Control and Complications Trial (DCCT) revealed that maintaining β‐cell c‐peptide secretion is associated with a reduced incidence of complications in T1DM.^18^ C‐peptide, the cleavage product of pro‐insulin, was believed to be inert for many years. It has been recently demonstrated that c‐peptide exerts several positive actions at different levels: on nerves, vasculature, smooth muscle relaxation, kidney function and bone.^19^ In the skeletal muscle, C‐peptide administration increases the glucose utilization through the stimulation of glucose transport.^19^ A recent preclinical study also suggests that C‐peptide administration modulates the body composition, especially lean mass.^20^ In that study, the diabetic control group rats (not treated with C‐peptide) underwent an 11% decrease, and the diabetic plus C‐peptide group (C‐peptide‐treated) had a 7% decreases in the body weight. However, the primary objective of that study was to test the effects of continuous C‐peptide infusion on bone of adult T1DM male rats with C‐peptide deficiency.^20^ The direct effects of the C‐peptide on skeletal muscle and on atrophy‐related genes expression have never been studied.

The present study represents the completion of our previous study^20^ and aims to fill a gap between research on insulin C‐peptide and the treatment of skeletal muscles loss. Specifically, we tested whether a 6‐week continuous administration of C‐peptide would prevent loss of skeletal muscles and modulate the expression of key regeneration and atrophy‐related genes in STZ‐induced diabetic rats.

## Materials and methods

### Animals

This work was conducted according to the European guidelines (2010/63/EU) for reducing animal suffering and with the approval of the Ethics Committee for Experimental Animals Welfare of the University Magna Grecia, Catanzaro (Auth. 10/01/2018), and the Italian Ministry of Health (Auth. No 353/2018‐protocol ADEAB.16, Auth. 9/05/2018).

In this study, twenty‐three, 4‐month‐old male Wistar rats (Charles River Laboratories) (400–500 g) were used, as previously described.^20^ The rats were fed with a commercial standard diet containing 1% calcium, 0.7% phosphorus (of which 0.4% nonphytate phosphorus), Ca/P 3:1 and 150 IU of vitamin D3 per 100 g. All rats were individually housed in polycarbonate cages with an ad libitum access to pellets of standard rodent diet, as well as bottles of tap water. The environmental conditions were characterized by a 12‐h/12‐h light/day cycle, 24 ± 1°C temperature and 55 ± 10% humidity.

### Study design

The rats were randomly divided into three groups^20^: normal control group (not treated)—CTR; diabetic control (not treated)—D‐CTR; diabetic plus C‐peptide (C‐peptide‐treated)—C‐PEP.

**Figure 1:**
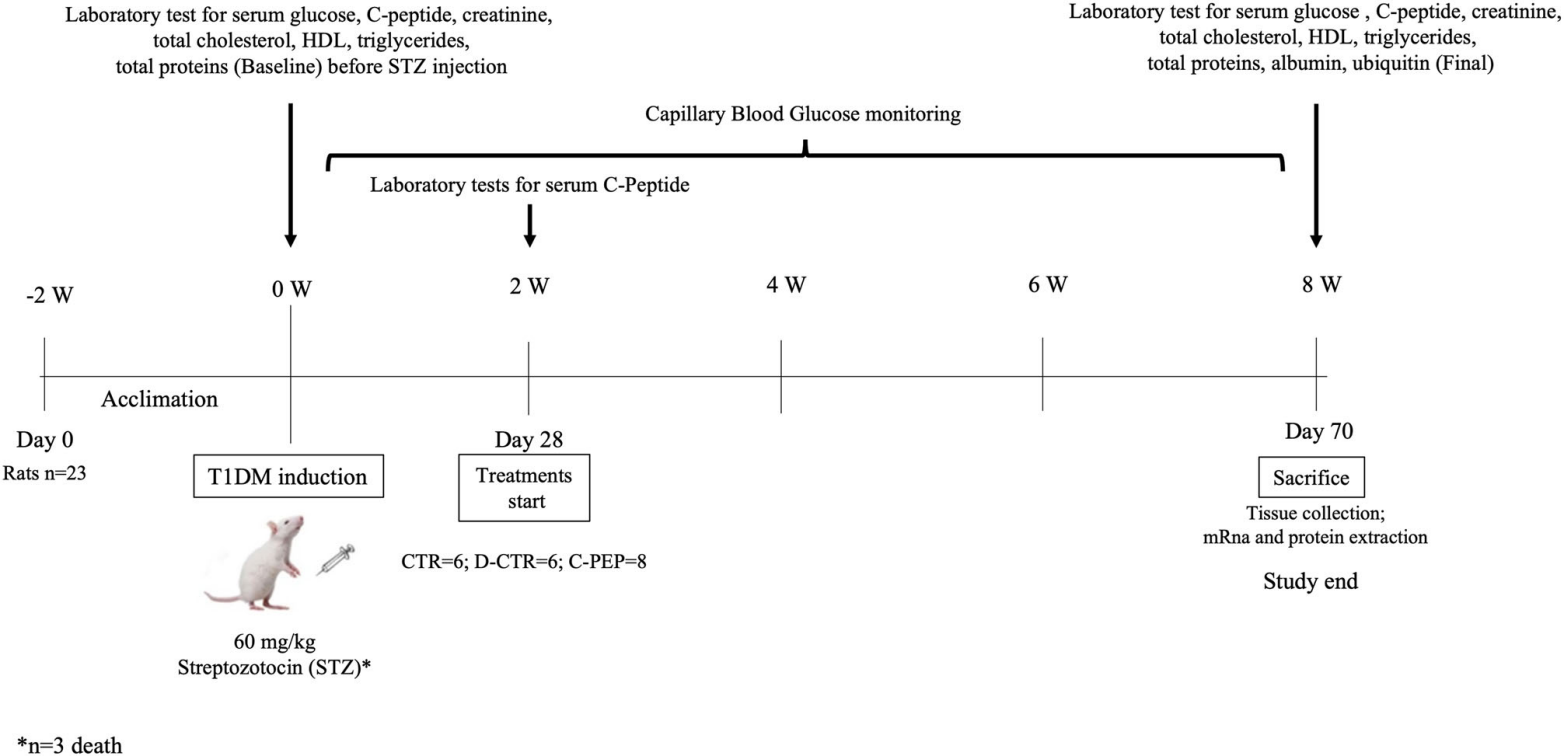
Experimental design of the study.

The experimental study design is showed at *Figure* *1*.

Diabetes was induced by a single intraperitoneal injection of STZ (60‐mg/kg body weight). Details on the STZ injection were previously reported.^20^ Briefly, STZ was dissolved in ice‐cold citrate buffer (0.05‐M citric acid, pH 5.1) and injected immediately within a few minutes to avoid degradation. Animals were considered diabetic only if their blood glucose levels were >300 mg/dL (16.6 mmol/L) 2 weeks after STZ injection. To prevent ketosis, a moderate dose of rapid and intermediate insulin was administered (Humalog and Humulin I, Eli Lilly Italia S.p.A.) when blood sugar levels were higher than 400 mg/dL to maintain a glucose level below 350 mg/dL.

On Day 28 (2 weeks after STZ injection), the rat C‐peptide (>95% purity, reversed‐phase high‐performance liquid chromatography) was delivered in C‐PEP group, via subcutaneously implanted osmo pumps (Alzet 2006, Alza, Palo Alto, CA) delivering 72 nmol · kg^−1^ · 24 h^−1^ ^20^ for 6 weeks,^20^ while the D‐CTR group was sham‐operated.^21^ Glucose concentration was monitored daily. The animals were euthanized after 6 weeks by isoflurane inhalation.

After the induction of diabetes, a total of three animals near death were immediately euthanized.

### Blood measurements

The blood samples were obtained after 2 weeks of acclimation (baseline assessment before STZ injection) and at end of the study (*Figure* *1*). All serum samples were stored frozen at −20°C until assayed. A volume of around 900 μL of whole blood was collected into untreated Eppendorf tubes and centrifuged for 15 min at 1500 × *g* at 4°C. The serum was then collected and stored at −80°C for later analysis. Serum glucose, creatinine, total proteins and lipids were analysed by Roche Module COBAS 8000 (Roche Diagnostics, Indianapolis, IN, USA). Serum C‐peptide was assessed with an enzymatic colorimetric test by ELISA kit (Crystal Chem Inc., USA).^20^ All measurements were performed in duplicate.

### Tissue sampling and skeletal muscle weight

Skeletal muscles were obtained post‐mortem, dissected and cleaned of other adherent tissues. Extensor digitorum longus (EDL), gastrocnemius (GC), tibialis anterior (TA) and soleus (SO) muscles were removed from the hindlimbs of rats and weighted. These muscles are commonly used for research of skeletal muscle in rodents. The GC, EDL and TA were chosen because they contain predominantly fast‐twitch type II fibre muscle, while SO muscle contains predominantly slow‐twitch type I fibre muscle. Muscle mass was evaluated by the muscle weight assessed immediately after sacrifice. The left leg muscle tissues were, then, frozen and stored at −80°C until processed but only those from a subgroup of eight rats were available for this study (CTR, *n* = 3; D‐CTR, *n* = 2; C‐PEP, *n* = 3). The right leg muscle tissues from all the rats were fixed with 4% paraformaldehyde for haematoxylin and eosin (H&E) staining and stored at +4°C.^22^

### Ubiquitin Elisa kit

Serum and muscular ubiquitin were assessed with an enzymatic colorimetric test by ELISA kit (MyBioSource, Inc., USA).

For serum sample, before dosage, the sample was centrifuged for 20 min at 1000 × *g* and stored at −80°C. At dosage moment, 10 μL from each serum sample rat (*n* = 6, CTR group; *n* = 6, D‐CTR group; and *n* = 8, C‐PEP group) were run in triplicate.

Muscle tissues from gastrocnemius and soleus were weighed, minced and homogenized in phosphate‐buffered saline on ice. Samples were then sonicated and centrifuged for 5 min at 5000 × *g*, and the supernatant was collected. Ten microliter from each sample rat were run in triplicate (*n* = 3, CTR group; *n* = 2, D‐CTR group; and *n* = 3, C‐PEP group). Evaluation of ubiquitin concentrations was based upon a colorimetric assay where the colour intensity was measured at an absorbance of 450 nm. The concentrations were determined by subtracting the background value and using the standard calibration curve equation.

### RNA extraction and quantitative reverse transcriptase‐PCR

Samples from the gastrocnemius and soleus were extracted according to the TRIzol isolation reagent protocol (Invitrogen, USA),^23^ and the quality and quantity of RNA were evaluated by measuring the absorbance at 260 and 280 nm on a spectrophotometer. Two micrograms of total RNA were reverse transcribed using High‐Capacity cDNA Reverse Transcription Kit. Quantitative reverse transcriptase (RT)‐PCR was performed in triplicates with 50‐ng cDNA template using SYBR green master mix with gene‐specific primers (*Table* *S1*). The reaction conditions were as follows: 40 cycles of initial denaturation temperature at 95°C for 15 s followed by annealing at 60°C for 15 s and extension at 72°C for 30s, and product specificity was analyzed by melt curve analysis. Data were compared between samples according to a comparative threshold cycle (2 − ΔΔct) method and normalized to β‐actin. Data are expressed as fold change over control.

### Protein extraction (markers of differentiation, regeneration and atrophy) and western blot analysis

For muscles protein extraction, ~50 mg of tissue were homogenized in liquid nitrogen and lysed in 100‐μL ice‐cold lysis buffer (100‐mM Trizma base pH 7.6, 300‐mM KCl, 1% Triton X‐100, and 10‐μL/mL freshly added protease and phosphatase inhibitor cocktails) and centrifuged at 20 000 g for 20 min at 4°C, and supernatant were collected. A total of 2 μL of the supernatant was used to determine the total protein concentration by Bradford assay (Quick Start Bradford 1 × Dye reagent, Bio‐Rad, USA) using bovine serum albumin (BSA) as a standard (Quick Start bovine serum albumin standard, Bio‐Rad, USA). Proteins were heat denatured for 5 min at 95°C in sample‐loading buffer, and 20 μg of protein lysate was resolved by sodium dodecyl sulphate‐polyacrylamide gel electrophoresis and transferred to nitrocellulose membranes (Amersham, UK). Membranes were blocked with Tris/HCl (pH 7.6) containing 0.1% Tween 20 and 5% BSA for 2 h and incubated overnight at 4°C with shaking with primary antibody Anti‐Fbx32 (ab168372, Abcam, UK), anti‐Murf1 (PA5‐76695, Thermo Fisher Scientific, USA), anti‐myosin heavy chain (MyHC) (#05‐716, Merck, Germany), anti‐MyoD1 (SAB1410813, Sigma Aldrich, USA), anti‐p extracellular signal‐regulated kinase (Erk)1/2 (9101), rabbit anti‐pAkt (9271S), anti‐SQSTM1/p62 (M162‐3), anti‐p 5′ AMP‐activated protein kinase (Ampk) (500815). Membranes were then washed in Tris‐buffered saline (pH 7.6) with 0.1% Tween 20 and incubated with horseradish peroxidase‐conjugated anti‐rabbit (Bio‐Rad, USA) or anti‐mouse (Bio‐Rad, USA) IgG secondary antibody for 1 h at room temperature with shaking. Bound antibody was visualized using the chemiluminescent kit (ECL WB Detection, Bio‐Rad, USA); immunoblot scanning and analyses were performed using a ChemiDoc Imaging System. Quantification of the bands was performed using the ImageJ software (NIH, Bethesda, MD, USA).

### Histological analysis of the muscle fibres

Sections of GC, SO, TA and EDL muscles were collected and fixed in 4% formalin for 24 h and paraffin embedded for morphological analysis. The muscle fibre diameters reveal a clear tendency to increase along the muscle, from proximal to distal compartments. The best consistency is achieved if mid‐belly sections are analyzed. For the assessment of tissue morphology, muscles samples were, thus, dissected from the widest part of the muscle. Samples from GC (both medial and lateral) were dissected where the muscle bellies are largest in diameter. Five‐micrometre thick muscle sections were stained with H&E and viewed at room temperature under a microscope (Leica DM 1000 LED) with a digital camera (LEICA ICC50 W) with 20X magnification. Measurements such as the cross‐sectional area (CSA), minimum Feret's diameter and perimeter were determined using ImageJ software thus allowing automated image acquisition, image analysis and storage.^24^ In order to determine all the paraments, a total of 200 to 300 fibres per muscle in each rat for a total of 10 images each rat were analysed. All analyses and measurements were performed in blinded fashion by the same observer (S. M.) to avoid bias in the results. Three representative images were randomly selected within the muscles. The distribution percentage of each morphological parameter (in bins) was determined using the GraphPad Prism software.

### Statistical analysis

Data are reported as mean ± standard deviation (*SD*). ANOVA test was used to compare the means between groups (as body weight, muscle mass, glucose, ubiquitin and other laboratory parameters). Changes from baseline to the end of the study (within group variation in body weight, glucose, total proteins, triglycerides and C‐peptide) were assessed using a paired Student's *t*‐test (two tailed). All the serum parameters were adjusted for the baseline body weight (body weight assessed after the acclimation period) by a general linear model with Bonferroni correction as post hoc analysis. All comparisons were performed using SPSS 25.0 for Windows (IBM Corporation, New York, NY, USA).

For genes and proteins expression, data resulted from a mean of at least three independent experiments and were analyzed with GraphPad Prism 5.0 software using a two‐tailed Student's *t*‐test. Significant differences were assumed to be present at *P* <  0.05 (two‐tailed).

## Results

### Effect of C‐peptide administration on skeletal muscle mass and body weight

The skeletal muscle mass (in terms of muscle weight) was recorded at the time of sacrifice. D‐CTR animals displayed significantly different skeletal muscle weight (considering the sum of each skeletal muscle) compared with the CTR and C‐PEP rats (CTR vs. D‐CTR, *P* < 0.001; C‐PEP vs. D‐CTR, *P* = 0.03; *Table* *1*), exhibiting a significantly lower muscle weight than CTR and C‐PEP rats.

**Table 1:** Tissues and muscles weight at the end of the study.

Variable	CTR^a^	D‐CTR^b^	C‐PEP^c^	*P*	Post hoc
	*n* = 6	*n* = 6	*n* = 8		
Gastrocnemius (g)	2.5 ± 0.15	2.08 ± 0.53	2.48 ± 0.16	0.042	a vs. b *P* = 0.025 b vs. c *P* = 0.035
Gastrocnemius(g;adj)	2.74 ± 0.14	2.05 ± 0.11	2.36 ± 0.11	0.007	a vs. b *P* = 0.002 b vs. c *P* = 0.07
Soleus (g)	0.27 ± 0.04	0.24 ± 0.02	0.28 ± 0.01	0.1	b vs. c *P* = 0.037
Soleus (g;adj)	0.27 ± 0.04	0.24 ± 0.03	0.28 ± 0.03	0.13	b vs. c *P* = 0.06
Tibiale (g)	0.98 ± 0.09	0.87 ± 0.09	0.98 ± 0.08	0.08	b vs. c *P* = 0.045
Tibiale (g;adj)	1.04 ± 0.1	0.87 ± 0.08	0.95 ± 0.09	0.016	a vs. b *P* = 0.006 b vs. c *P* = 0.09
EDL (g)	0.23 ± 0.01	0.21 ± 0.01	0.22 ± 0.01	0.015	a vs. b *P* = 0.004
EDL (g;adj)	0.24 ± 0.02	0.21 ± 0.01	0.22 ± 0.01	0.041	a vs. b *P* = 0.014
All muscles (g)	1.5 ± 0.09	1.32 ± 0.14	1.48 ± 0.08	0.018	a vs. b *P* = 0.009 b vs. c *P* = 0.017
All muscles (g; adj)	1.59 ± 0.09	1.31 ± 0.09	1.43 ± 0.1	0.001	a vs. b *P* < 0.001 b vs. c *P* = 0.031
WAT (g)	3.25 ± 1.3	2 ± 0.5	1.6 ± 0.5	0.01	a vs. b *P* = 0.018 a vs. c *P* = 0.004
WAT (g;adj)	3.39 ± 1	1.92 ± 0.9	1.57 ± 0.9	0.03	a vs. b *P* = 0.026 a vs. c *P* = 0.011

*Note*: Each significant difference is adjusted for the baseline body weight with general linear model analysis.

Abbreviations: EDL, extensor digitorum longus; WAT, white adipose tissue.

^a^ CTR.

^b^ D‐CTR.

^c^ C‐PEP.

**Figure 2:**
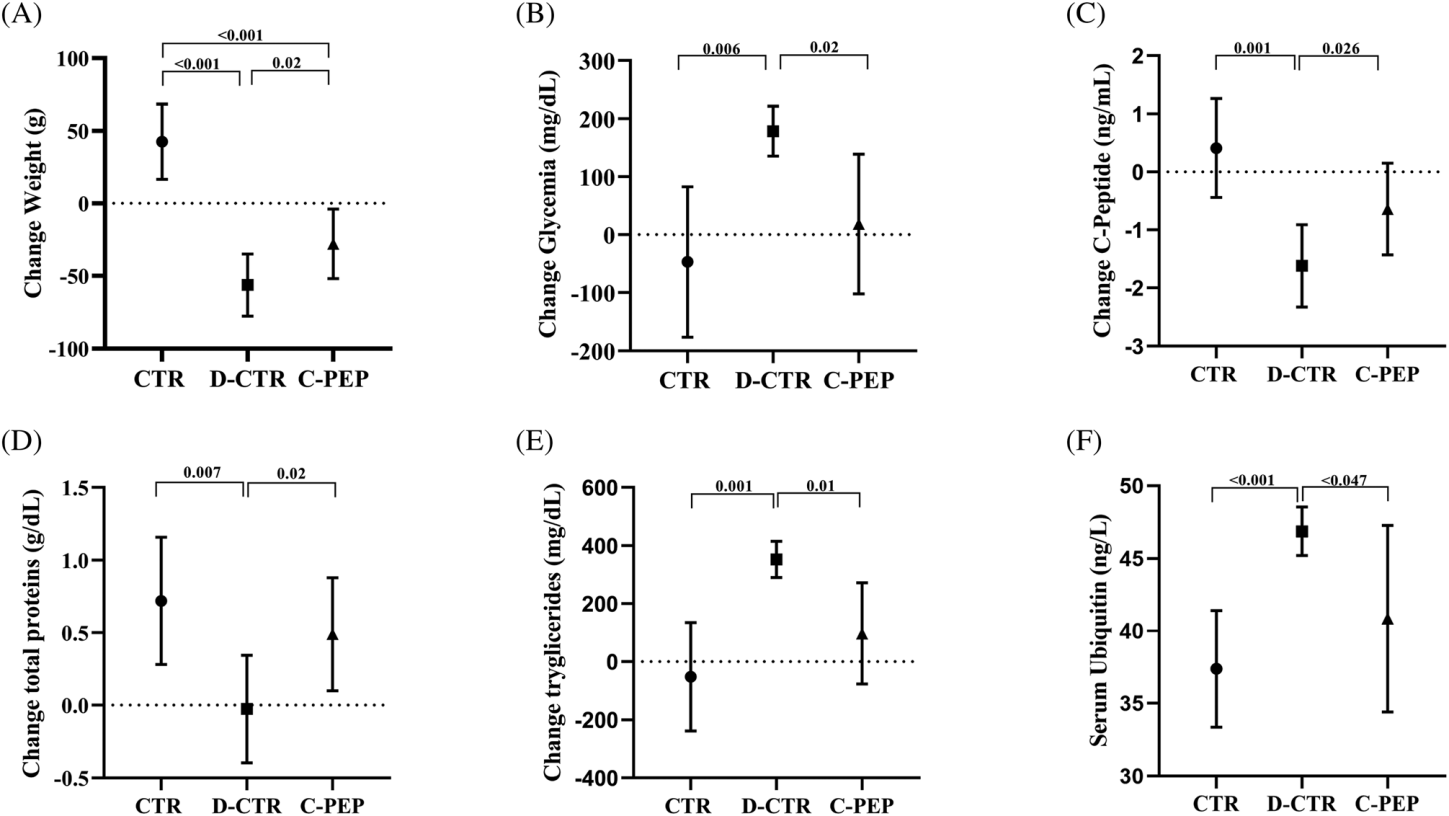
Clinical characteristics of rats according to intervention group. CTR groups (*n* = 6 rats), D‐CTR (*n* = 6 rats), C‐PEP (*n* = 8 rats). (A) Weight change, (B) glycaemia change, (C) C‐peptide change, (D) total proteins change, (E) triglycerides change and (F) serum ubiquitin level (measured with Elisa Kit). Data are represented as means ± *SD*. Note: Each significant difference is adjusted for the baseline body weight with general linear model analysis.

D‐CTR animals displayed a lower weight of the GC, SO, TA and EDL, considered individually, than CTR and C‐PEP rats (unadjusted *P* = 0.03; *P* = 0.03; *P* = 0.04; *P* = 0.004, respectively). C‐peptide prevented body weight loss (C‐PEP vs. D‐CTR *P* = 0.02 *Figure* *2A*, *Table* *1*).

### Effect of C‐peptide administration on serum parameters and ubiquitin

Three animals died after the induction of diabetes due to glycaemic decompensation. All of them were in the D‐CTR group. C‐peptide administration prevented serum protein reduction and reversed hyperglycaemia and hypertriglyceridaemia in C‐PEP rats compared with D CTR (C‐PEP vs. D‐CTR *P* = 0.02; 0.02; 0.007; 0.01; respectively, *Figure* *2B,E* and *Table* *S2*).

D‐CTR animals presented significantly higher concentration of ubiquitin in the serum compared with the C‐PEP and CTR animals (C‐PEP vs. D‐CTR *P* = 0.02 CTR vs. D‐CTR *P* = 0.01; *Figure* *2F*). C‐PEP rats in SO showed lower protein expression levels of ubiquitin compared with both CTR and D‐CTR (*P* = 0.01; *P* = 0.04, respectively *Figure* *S1*). Although not significant, the expression levels of ubiquitin in GC was lower in the C‐PEP rats compared with D‐CTR (*P* = 0.07, *Figure* *S1*).

### Effect of C‐peptide on markers of differentiation, regeneration and atrophy

mRNA expression of *MyoD1* in the GC, but not in TA, EDL and SO, was higher in the C‐PEP group than in D‐CTR and CTR rats (C‐PEP vs D‐CTR *P* = 0.003; CRT vs D‐CTR *P* = 0.001; *Figure* *S2*).

The mRNA levels of *Atrogin‐1*, *MuRF‐1* and *Traf6* in the skeletal muscles were then analysed (*Figure* *S2*). mRNA expression of *Atrogin‐1* and *Traf6* in the TA, EDL and GC was lower in the C‐PEP group than in D‐CTR rats (*Figure* *S2*; *Atrogin‐1*: C,H,O; *Traf6*: D,I,P) while these mRNA expressions did not differ between groups in SO (*Figure* *S2* R‐V).

**Figure 3:**
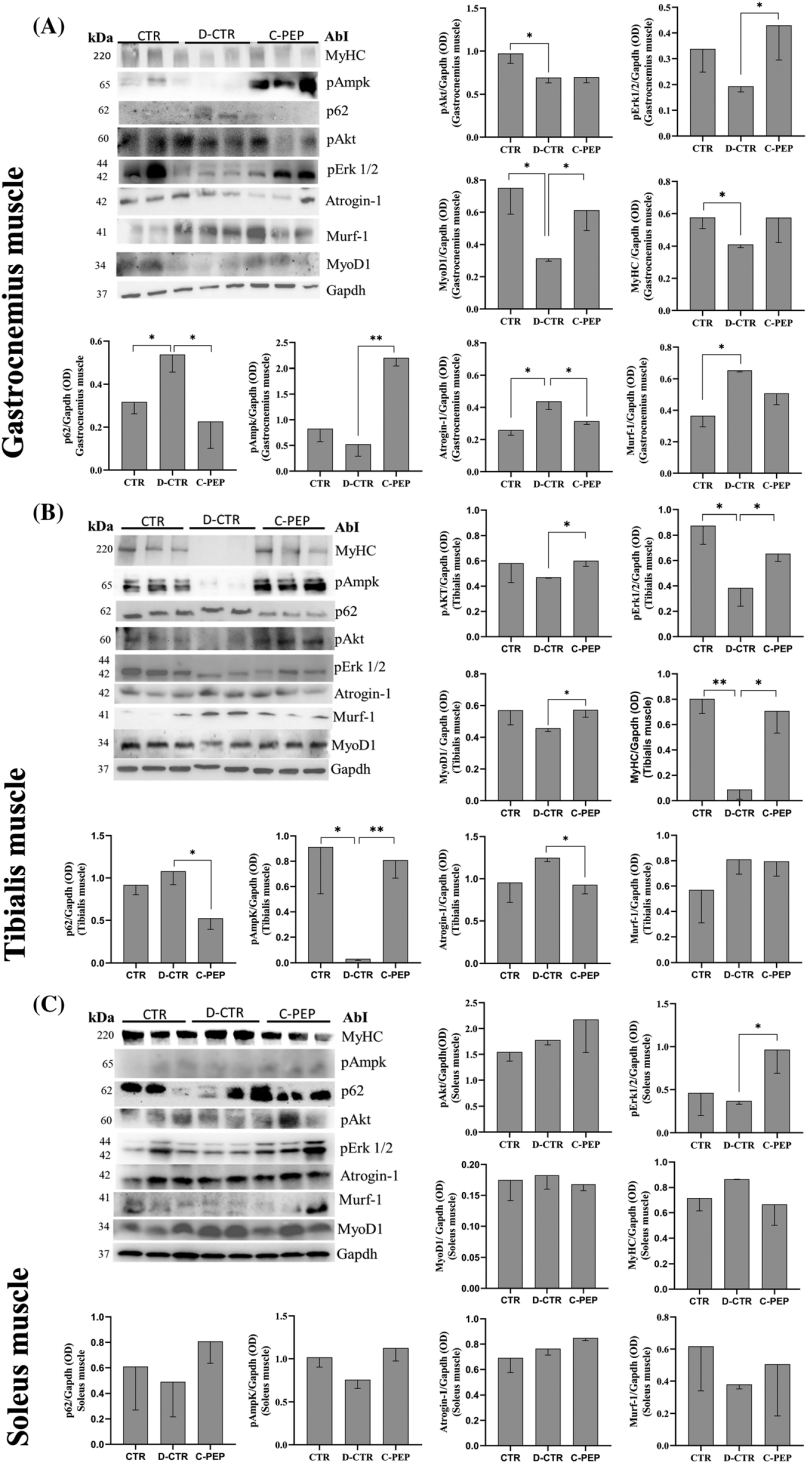
C‐peptide increases pErk1/2, pAmpK, MyoD1 and reduces p62 and Atrogin‐1 protein expression level in gastrocnemius (GC) muscle (A); in tibialis anterior (TA) muscle, C‐peptide increases pAkt, pErk1/2, pAmpK, MyoD1, MyHC and reduces p62 and Atrogin‐1 protein expression level (B) and increases pErk1/2 protein expression level in soleus (SO) muscle (C). All proteins expression levels were analyzed by Western blotting, with specific antibodies. Data are represented as mean ± *SD* of three independent experiments, in a subgroup of rats (*n* = 3 [CTR], *n* = 2 [D‐CTR] and *n* = 3 [C‐PEP]). Statistical analysis: Student's *t*‐test *P < 0.05 **P < 0.01.

The protein expression level of MyoD1 in GC and TA was higher in the C‐PEP group than in D‐CTR rats (*P* = 0.04, *P* = 0.04; *Figure* *3A,B*) and did not differ significantly compared with CTR rats. The protein expression of Atrogin‐1 in GC and TA was lower in the C‐PEP group than in D‐CTR rats (*P* = 0.02, *P* = 0.03; *Figure* *3A,B*) and did not differ significantly compared with CTR rats. The protein expression levels of MyHC in TA and EDL was higher in the C‐PEP group than in D‐CTR rats (*P* = 0.02, *P* = 0.02, respectively; *Figures* *3B* and *S3*). Although not significant in GC, there was a trend towards lower MuRF‐1 and higher MyHC in C‐PEP rats than in D‐CTR rats (*Figure* *3A*).

### Effect of C‐peptide on autophagy and atrophy pathways

In GC, TA, SO and EDL, the protein expression of pErk1/2 was higher in C‐PEP group than D‐CTR rats (*P* = 0.03; *P* = 0.02; *P* = 0.01; and *P* = 0.02, respectively) (*Figures* *3A–C* and *S3*).

In the GC and TA, the pAmpk expression was higher in the C‐PEP group than the D‐CTR (*P* = 0.002 and *P* = 0.005, respectively; *Figure* *3A,B*). Furthermore, in GC, TA and EDL, p62 was lower in C‐PEP than D‐CTR group (*P* = 0.04; *P* = 0.02; and *P* = 0.002, respectively; *Figures* *3A,B* and *S3*). The expression of Akt was higher in the C‐PEP group than D‐CTR only in the TA muscle (*P* = 0.003; *Figure* *3B*).

### Effect of C‐peptide administration on WAT and lipids metabolism

**Figure 4:**
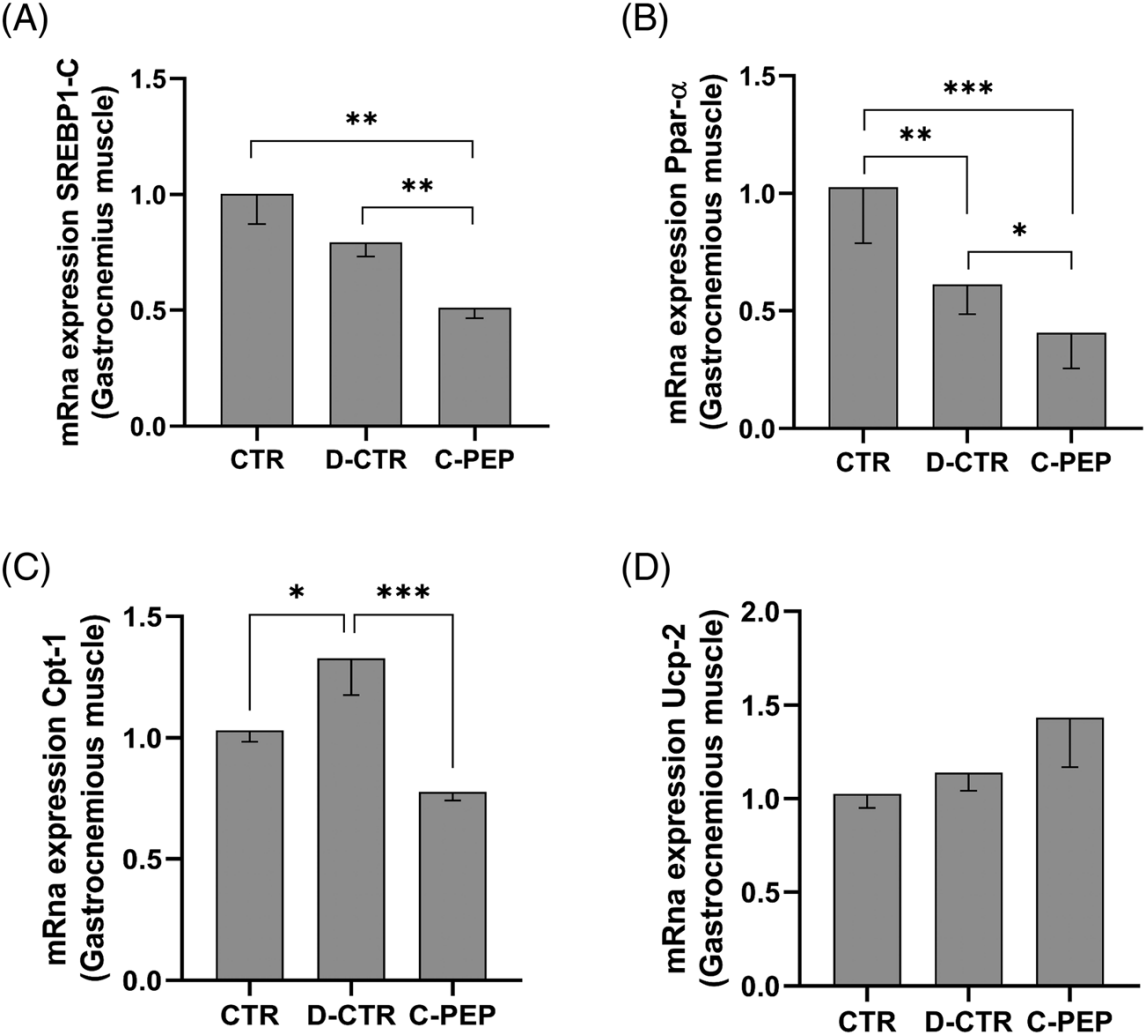
C‐peptide reduces, in gastrocnemius (GC) muscle, Srebp‐1c, Ppar‐α and Cpt‐1 mRna expression. (A) Srebp‐1c, (B) Ppar‐α, (C) Cpt‐1 and (D) Ucp‐2 mRna expression were measured by RT‐PCR. Data were analyzed using the 2‐ΔΔCq method and normalized to β‐actin. Data are represented as means ± *SD* of three independent experiments, in a subgroup of rats (*n* = 3 [CTR]), *n* = 2 [D‐CTR] and *n* = 3 [C‐PEP]). Statistical analysis: Student's *t*‐test *P < 0.05, **P < 0.01, ***P < 0.001.

In C‐PEP and D‐CRT rats, as expected, WAT was significantly lower than CTR rats (*Table* *1*). *Figure* *4A–C* shows, however, a lower *Srebp‐1c*, *PPAR‐α* and *Cpt‐1* mRNA expression in the gastrocnemius of C‐PEP rats compared with the D‐CTR group (*P* = 0.002; *P* = 0.01 and *P* < 0.001, respectively, *Figure* *4*) while *Ucp2* expression did not differ between the groups (*Figure* *4D*).

### Effect of C‐peptide on fibre cross‐section areas and other parameters

**Figure 5:**
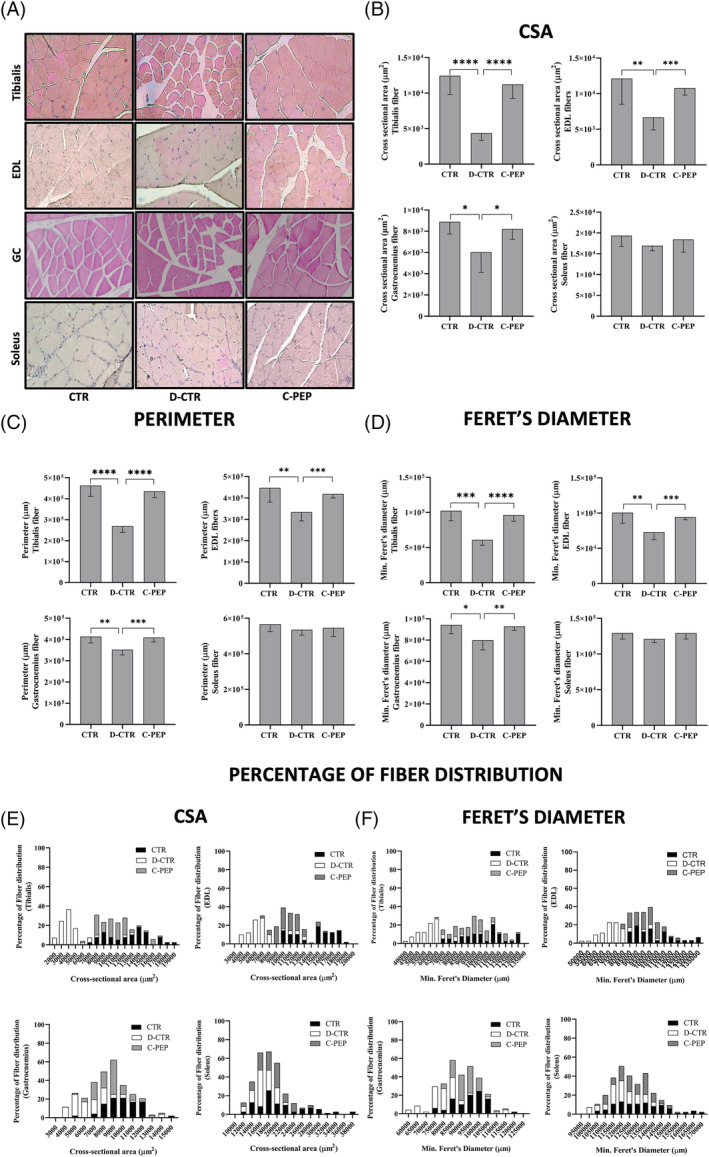
In gastrocnemius (GC), tibialis anterior (TA) and extensor digitorum longus (EDL) muscle fibres, C‐peptide improves the cross‐sectional area (CSA), min. Feret's diameter and perimeter. (A) Representative images of haematoxylin and eosin staining of the TA, EDL, GC and SO muscles (×20 magnification). (B) CSA of TA, EDL, GC and soleus (SO) muscles. (C) Perimeter of TA, EDL, GC and SO muscles; (D) Min. Feret's diameter of TA, EDL, GC and SO muscles; (E, F) Percentage distribution of fibres' CSA and min. Feret's diameter of TA, EDL, GC and SO. Data are represented as means ± *SD* (*n* = 6 [CTR], *n* = 6 [D‐CTR] and *n* = 8 [C‐PEP]). Statistical analysis: Student's *t*‐test *P < 0.05, **P < 0.01, *** *P* < 0.001 and ****P < 0.0001.

During the experimentation period, the C‐PEP group reduced CSA by 6.6% while the D‐CTR underwent a 39.5% compared with the CTR animals in the GC (*Figure* *5B*, *P* = 0.02). The CSA of TA and EDL muscles was reduced, in the C‐PEP, by 10% and 11%, respectively, while there was a reduction of 65% and 45% in the D‐CTR group compared with CTR animals (*Figure* *5B*, both *P* < 0.0001). Similar results were obtained with the other parameters (minimum Feret's diameter and perimeter) (*Figure* *5C,D*). The frequency distribution of CSA, perimeter and minimum Feret's diameter was consistent with the above results (*Figures* *5E,F* and *S4*). In SO, CSA, minimum Feret's diameter and perimeter did not differ between the groups (*Figures* *5* and *S4*).

## Discussion

Despite its relevant role as a metabolic organ, there are few preclinical and clinical studies investigating changes in skeletal muscle during T1DM. In the present study, even though blood glucose was corrected, insulin administration did not prevent the increase in muscle proteolysis and the rise in UPS component mRNAs in diabetic rats (*Figure* *4B*). Our histological assessment revealed that D‐CTR rats experienced a decreased muscle mass and fibre CSAs in the GC, TA, EDL muscles (*Table* *1* and *Figure* *5*). After 42 days, mean GC fibre CSAs decreased by 39.5% and 6.6% in D‐CTR and C‐PEP rats, respectively (*Figure* *5B*). Furthermore, we found only a 10% and 11% reduction in the CSA of TA and EDL muscles in the C‐PEP but a 65% and 45% reduction in these parameters in the D‐CTR group compared with CTR animals. Parameters such as the perimeter and minimum Feret's diameter showed a similar result (*Figure* *5C,D*).

C‐peptide, thus, prevented fibres atrophy in the GC, TA and EDL by obtaining a similar value in parameters as CSAs, perimeter and minimum Feret's diameter to that of CTR rats. This in an unprecedented finding. Normal myofibres have a polygonal shape and homogenous fibre areas. The SO muscle is composed of a higher proportion of slow twitch fibres than its synergists GC, TA and EDL, respectively. Because the most evident sign of sarcopenia in human is represented by the atrophy of the fast‐twitch fibres, our results would suggest that the C‐peptide represents a possible agent for the prevention of sarcopenia. However, in this study, we did not analyzed the different types and subtypes of fibres. Based on the finding that, in SO, CSA, minimum Feret's diameter and perimeter did not differ between the groups, we can thus assume that C‐peptide has a prominent effect on fast fibres. However, further studies are needed to better clarify the differential effect of C‐peptide among different types of fibres.

An important finding of this work is that, in GC and TA, the pAmpk expression was higher in the C‐PEP group than D‐CTR (*Figure* *3A,B*). Although not significant, in EDL, there was a trend towards higher pAmpk in C‐PEP rats than in D‐CTR rats (*Figure* *S3*). AMPK represents a key enzyme in regulating skeletal muscle mass and glucose uptake in muscles.^15^ Exercise‐induced AMPK activation has been shown to regulate nutrient supply as well as mitochondrial biogenesis in human skeletal muscle.^25^, ^26^ It has been already demonstrated that C‐peptide acts against hyperglycaemia‐induced endothelial damage through the stimulation of the AMPK pathway.^27^

AMPK regulates the mechanisms of atrophy possibly through modulating the UPS.^28^ We found that C‐peptide reduced the levels of serum ubiquitin for skeletal muscle mass and fibre size. Our findings also reveal, for what we believe is the first time, that C‐peptide may modulate muscle mass and glucose homeostasis by the pAmpk pathway. Diabetes is characterized by diabetic myopathy as a result of increased proteolysis and decreased protein synthesis which leads to muscle atrophy.^6^, ^7^ T1DM in murine models is usually studied by the induction of the diabetes by streptozotocin injection, which rapidly induces muscle wasting.^9^ This phenomenon is similar to that shown by T1DM patients upon withdrawal of insulin treatment. In T1DM animal models, UPS is activated which leads to protein degradation and a decrease in the weights of the muscles.^11^ Furthermore, due to its role in the endoplasmic (ER) stress, UPS is also crucial in the development of the common diabetic complications.^29^

Our results would suggest that the inhibition of the proteasomal activity by C‐peptide prevents muscle wasting or cachexia in T1DM.

Furthermore, it has been suggested that Ampk‐mediated modulation of FoxOs contributes to activating the autophagy systems in skeletal muscle cells.^30^ An activation of autophagy, which participates in the loss of muscle mass, has been demonstrated in streptozotocin‐induced diabetes.^15^ Consequently, a crosstalk is likely between proteasome‐dependent degradation and autophagy. In fact, ubiquitin is involved in both the degradation pathways UPS and autophagy. p62 is an autophagy adaptor in mammals that delivers ubiquitinated cargoes for autophagic clearance.^31^ The TRAF6‐binding domain interacts with TRAF6 proteins (an important E3 ubiquitin ligase) to trigger protein polyubiquitination.^31^ In CG, TA and EDL we find that p62 was lower in the C‐PEP than D‐CTR group (*Figures* *3* *A, B* and *S3*). The mRNA expression of *Traf6*, in all these muscles, was lower in the C‐PEP group than in D‐CTR rats (*Figure* *S2*). Depletion of TRAF6 resulted in a drastic improvement in the fibre CSA in a mice model of skeletal muscle wasting.^32^ p62 and TRAF6 would seem to represent promising strategic targets for the treatment of muscle loss.^31^ However, the relative contribution of these two proteins to muscle atrophy needs to be better determined in the future.

Inhibition of ubiquitin expression is crucial but not sufficient by itself to inhibit muscle proteolysis (*Figure* *S1* *A*). In fact, the SO muscle expressed less ubiquitin than the GC but, on histological analysis, C‐peptide prevented muscle damage only in GC, TA and EDL (*Figure* *5A–E*). One prominent subset of the pro‐cachectic molecule includes two key E3 ligases, namely, MURF1 and Atrogin‐1, which are, in turn, highly upregulated by FOXO3.^14^, ^16^ In the present study, the mRNA levels of *Atrogin‐1*, *MuRF‐1* and *Traf6* did not differ between groups in SO (*Figure* *S2*). We found that mRNA expression of *Atrogin‐1* and its protein assessed in the GC, TA and EDL were lower in the C‐PEP group than in D‐CTR rats (*Figures* *3A,B*, S2 and S3). These molecules mediate sarcomeric breakdown and inhibition of protein synthesis in skeletal muscle where they tag lysine residues of proteins with poly‐ubiquitin chains.^14^, ^16^ These mechanisms, activate proteolysis in response to catabolic stress during diabetes.^14^, ^16^ In line with our findings, several studies have reported changes in muscle E3 ligases and ubiquitinated proteins in skeletal muscle under acute hyperglycaemia.^33^, ^34^

In line with a study of Zhong et al.,^35^ we also found that C‐peptide signal transduction involves phosphorylation of ERK1/2 and a parallel activation of Akt. An anti‐apoptotic role of Akt/PKB stimulation has been demonstrated.^36^ The massive muscle fibre degeneration during T1DM may reflect severe defects in the proliferation and/or survival programmes of myocytes, possibly related to the lack of periodical stimulation by C‐peptide. These findings are important because they confirm that signals transmitted by C‐peptide are likely to play a crucial role in maintaining the muscle mass also by the ERK1/2 pathway.

The present study has important clinical implications. Cancer cachexia represents the primary cause of death for 20–30% of all cancer patients.^37^ The ubiquitin‐dependent system, Atrogin‐1 and MuRF‐1 are upregulated in cancer cachexia mice.^37^ These findings imply that the potential mechanism underlying the muscle wasting of cancer cachexia could be similar to D1TM, and thus, our study could suggest that C‐peptide may be a potential agent for treating muscle atrophy also induced by cancer cachexia. Future research could focus on the role of C‐peptide in the early stage of muscle atrophy in humans affected by T1DM and in cancer cachexia.

There are currently no approved drugs to treat muscle loss in patients with diabetes. Between 2015 and 2050, the proportion of the world's population over 60 years will nearly double; thus, there is corresponding interest in the development of drugs to combat muscle wasting in individuals with diabetes and sarcopenia in order to improve the quality of life and reduce healthcare costs. C‐peptide treatment seems to exert a fascinating preventive action in atrophied muscles of diabetic animals. Human studies are needed to clarify its contribution in reversing the muscle degeneration in individuals with T1DM. The *MyoD1* gene is expressed exclusively in skeletal muscle. MyoD1 is important for postnatal myoblasts differentiation, muscle regeneration and mitochondrial biogenesis.^38^ Indeed, the stimulation of MyoD1 alone is sufficient to trans‐differentiate fibroblasts and other cell types to muscle.^39^ Thus, our findings may suggest that C‐peptide, via MyoD1, is not only responsible for the transcription of the sarcomeric genes involved in contractile function, but it also supports energy fuelling. The benefits of MyoD1 stimulation in skeletal muscle by C‐peptide raise novel possibilities for therapeutic approaches for the treatment of muscle wasting in humans.

Interestingly, we found that the change in body weight of the C‐PEP rats was lower than D‐CTR rates, thus indicating that C‐peptide modulates body composition. In our study we thus measured WAT (*Table* *1*). However, WAT mass did not differ between C‐PEP and D‐CTR rats. To better understand the involvement of C‐peptide on the metabolism of fat mass, TG and VLDL and lipid synthesis, we assessed Srebp‐1c, PPAR‐α and Cpt‐1 expression. Both Srebp‐1c and PPAR‐α are transcription factors that regulate lipid metabolism in the liver. Srebp‐1c also regulate the expression of Cpt‐1. PPAR‐α increases significantly in atherosclerosis, as confirmed by the present study. We found a lower *Srebp‐1c*, *PPAR‐α* and *Cpt‐1* mRNA expression in the gastrocnemius of C‐PEP rats compared with the D‐CTR group. In our model, the high triglyceride load likely causes PPAR‐α activation, but C‐peptide could revert this mechanism. Accordingly, triglyceridaemia was significantly reduced in C‐PEP compared with D‐CTR rats after 42 days, suggesting a potential action of C‐peptide also on both lipid and liver metabolism. However, further studies are needed to better clarify these latter points.

Our study has some limitations. We used only male rats and did not use rodent models of women with T1DM and muscle loss. Furthermore, one disadvantage with chemically induced diabetes is that the chemicals can be toxic for other organs of the body. Changes in P450 isozymes in the liver, kidney, lung, intestines, testis and brain have been reported after administration of STZ but not in muscle. Chemically induced diabetes thus remains a simple and relatively cheap model of diabetes in rodents. In the present study, we used 4‐month old rats that reached both the skeletal and muscle maturity. This feature may be considered an important strength of our study.

Unfortunately, due to an electrical problem and the consequent partial loss of rats organs and tissues, we performed western blot and RT‐PCR only in a subgroup of rats (CTR n.3, D‐CTR n.2 and C‐PEP n.3). However, the potential clinical significance of C‐peptide in muscle wasting is highlighted by all the present results.

## Conclusions

We demonstrated, for the first time, that C‐peptide administration in rats could protect several skeletal muscles as GC, TA and EDL from atrophy induced by T1DM. Taken together, our data suggest that targeting the UPS, Ampk, MyoD‐1 and muscle‐specific E3 ubiquitin ligases such as Atrogin‐1 and Traf6 may be an effective strategy for molecular and clinical intervention in the muscle wasting pathological process in T1DM.

## Conflict of interest

The authors declare that they have no relevant conflict of interest.

## Supporting information


**Figure S1:** Levels of ubiquitin in SO were lower in the C‐peptide group than diabetic control rats. (A) Ubiquitin levels of SO (B) and GC were measured by Elisa Kit (*n* = 3 (CTR), *n* = 2(D‐CTR) and *n* = 3 (C‐PEP). Data are represented as the means ± SD. Statistical analysis: Student's t‐test * *P* < 0.05
**Figure S2.** C‐peptide increases MyoD‐1 mRna expression, in GC (M), downregulates Murf‐1 (B), UbC (E) Atrogin‐1 and Traf6 mRna expression in TA (C and D), EDL (H and I) and GC (O and P) muscles. Data are represented as the means ± SD (CTR (n.3 rats), D‐CTR (n.2 rats), C‐PEP (n.3 rats)). Statistical analysis: Student's t‐test * *P* < 0.05 **P < 0.01 and ***P < 0.001.
**Figure S3.** C‐peptide increases pErk1/2 and MyHC and decreases p62 protein levels on EDL muscle. All proteins expression levels were analyzed by Western blotting with specific antibodies. Data are represented as mean ± SD of three independent experiments, in a subgroup of rats (*n* = 3 (CTR), *n* = 2 (D‐CTR) and *n* = 3 (C‐PEP)). Statistical analysis: Student's t‐test * *P* < 0.05 ** *P* < 0.01
**Figure S4.** Percentage distribution of fibres' perimeters in GC, EDL, TA and SO. Data are represented as the means ± SD (*n* = 6 (CTR), *n* = 6 (D‐CTR) and *n* = 8 (C‐PEP).
