# Supplementary material for: Preventing muscle wasting: pro‐insulin C‐peptide prevents loss in muscle mass in streptozotocin‐diabetic rats
Source: J Cachexia Sarcopenia Muscle. 2023 Mar 6;14(2):1117–29. doi: 10.1002/jcsm.13210 (PMC10067479; doi:10.1002/jcsm.13210)
Supplement: Supplementary file 1 — Figure S1: Levels of ubiquitin in SO were lower in the C‐peptide group than diabetic control rats. (A) Ubiquitin levels of SO (B) and GC were measured by Elisa Kit (n = 3 (CTR), n = 2(D‐CTR) and n = 3 (C‐PEP). Data are represented as the means ± SD. Statistical analysis: Student's t‐test * P < 0.05 Figure S2. C‐peptide increases MyoD‐1 mRna expression, in GC (M), downregulates Murf‐1 (B), UbC (E) Atrogin‐1 and Traf6 mRna expression in TA (C and D), EDL (H and I) and GC (O and P) muscles. Data are represented as the means ± SD (CTR (n.3 rats), D‐CTR (n.2 rats), C‐PEP (n.3 rats)). Statistical analysis: Student's t‐test * P < 0.05 **P < 0.01 and ***P < 0.001. Figure S3. C‐peptide increases pErk1/2 and MyHC and decreases p62 protein levels on EDL muscle. All proteins expression levels were analyzed by Western blotting with specific antibodies. Data are represented as mean ± SD of three independent experiments, in a subgroup of rats (n = 3 (CTR), n = 2 (D‐CTR) and n = 3 (C‐PEP)). Statistical analysis: Student's t‐test * P < 0.05 ** P < 0.01 Figure S4. Percentage distribution of fibres' perimeters in GC, EDL, TA and SO. Data are represented as the means ± SD (n = 6 (CTR), n = 6 (D‐CTR) and n = 8 (C‐PEP). [file JCSM-14-1117-s001.docx]

| *Supplemental Table 1. Real-Time primer sequences* | | |
| --- | --- | --- |
| **Gene** | **Forward** | **Reverse** |
| *Myod-1* | 5’-TGCTCTGATGGCATGATGGA-3’ | 5’-CCATCCCCATGAGTGCAGAC-3’ |
| *Atrogin-1* | 5’-GGCCTTCAAAGGTCTCACGA-3’ | 5’-GGTGAAAGTGAGACGGAGCA-3’ |
| *Murf-1* | 5’-ACCAAGGAAAACAGCCACCA-3’ | 5’-GCCTTGTTCTGTCTTCCCCA-3’ |
| *Traf-6* | 5’-ACTTGATCTCGGAGTGCTGC-3’ | 5’-CGTGACAGCCAAACACACTG-3’ |
| *UbC* | 5’-ACACCAAGAAGGTCAAACAGGA-3’ | 5’-CACCTCCCCATCAAACCCAA-3’ |
| *Srebp-1C* | 5’-CCAGCCTTTGAGGATAACCA-3 | 5’-TGCAGGTCAGACACAGGAAG-3 |
| *Ppar-α* | 5’-ATCCACGAAGCCTACC-3 | 5’-CACACCGTACTTTAGCAAG-3 |
| *Cpt-1a* | 5’-CCAGGCTACAGTGGGACATT-3 | 5’-GAACTTGCCCATGTCCTTGT-3 |
| *Ucp-2* | 5’-CAGAGCACTGTCGAAGCCTA-3 | 5’-GTATCTTTGATGAGGTCATA-3 |
| *Β-actin* | 5’-GCCCTGAGGCACTCTTCCA-3 | 5’-TTGCGGATGTCCACGTCA-3 |
|  |  |  |
| *Abbreviation. Myod-1 =Myoblast determination protein 1; Atrogin-1=1/muscle atrophy F-box; Murf-1= muscle ring-finger protein 1; Traf-6=TNF Receptor Associated Factor 6; UbC=Ubiquitin C; Srebp1-C= Sterol regulatory element-binding protein 1; Ppar-α=*  *Peroxisome proliferator-activated receptor alpha; Cpt-1a= carnitine palmitoyltransferase 1A; Ucp-2=Uncoupling Protein 2.* | | |

| Supplemental Table 2. *Baseline and final clinical characteristics of rats according to intervention group* | | | | | | | | | | |
| --- | --- | --- | --- | --- | --- | --- | --- | --- | --- | --- |
| **Variables** | **CTR^a^**  **(n=6)** | **D-CTR^b^**  **(n=6)** | **C-PEP^c^**  **(n=8)** | ***P* ***** | ***Post-hoc*** | **CTR^a^**  **(n=6)** | **D-CTR^b^**  **(n=6)** | **C-PEP^c^**  **(n=8)** | ***P* ***** | ***Post-hoc*** |
|  | *Basal* | | | | | *Final* | | | | |
| **Body weight (g)** | 483±44 | 554±46 | 570±41 | 0.007 | b vs a, p=0.029  c vs a, p=0.002 | 546±30 | 485±39 | 529±33 | 0.019 | a vs b, p=0.007  b vs c, p=0.029 |
| **Glucose (mg/dL)** | 106±43 | 131±31 | 124±19 | 0.39 | / | 102±21 | 303±151 | 115±54 | 0.02 | a vs b, p=0.002  b vs c, p=0.022 |
| **C-peptide (mg/dL)** | 1.2 ± 0.7 | 2.01 ± 0.7 | 1.6 ± 0.8 | 0.23 | / | 1.62 ± 0.4 | 0.39 ± 0.3 | 0.98 ± 0.3 | <0.001 | a vs b, p<0.001  a vs c, p=0.015  b vs c, p=0.006 |
| **Total cholesterol (mg/dL)** | 71.8 ± 14 | 96 ± 12 | 98 ± 13 | 0.015 | a vs b, p<0.008  a vs c, p=0.007 | 83 ± 19 | 114 ± 15 | 115 ± 19 | 0.02 | a vs b, p<0.009  a vs c, p=0.016 |
| **HDL** | 42.6 ± 4.3 | 61.9 ± 3.5 | 61.9 ± 3.5 | 0.009 | a vs b, p<0.004  a vs c, p=0.006 | 54.8 ± 14 | 64.5 ± 11 | 73 ± 12 | / | / |
| **Triglycerides (mg/dL)** | 85 ± 37 | 129 ± 37 | 132 ± 49 | 0.13 | / | 91 ± 36 | 472 ± 235 | 187 ± 68 | 0.001 | a vs b, p<0.001  b vs c, p=0.02 |
| **Creatinine (mg/dL)** | 0.33±0.03 | 0.37±0.04 | 0.45±0.01 | 0.33 | / | 0.40±0.02 | 0.46±0.02 | 0.39±0.02 | 0.29 | / |
| **Total proteins (g/dL)** | 5.8± 0.4 | 6.2 ± 0.3 | 6 ± 0.3 | 0.21 | / | 6.5 ± 0.4 | 6.1 ± 0.3 | 6.5 ± 0.2 | 0.07 | b vs c, p=0.047 |
| **Albumin (g/dL)** | / | / | / | / | / | 2.9 ± 0.2 | 2.7 ± 0.1 | 2.7 ± 0.1 | 0.02 | a vs b, p=0.015  a vs c, p=0.011 |
| **Rapid Insulin (U/day)** | / | / | / | / | / | / | 1.4±1.7 | 1.4±2 | 0.90 | / |
| **Intermediate**  **Insulin (U/day)** | / | / | / | / | / | / | 2.2±3 | 2±3 | 0.28 | / |
| *Note.* each significative difference is adjusted for the baseline body weight (except glucose) with General Linear Model analysis (***). Abbreviation: a=CTR, b=D-CTR, c=C-PEP. | | | | | | | | | | |


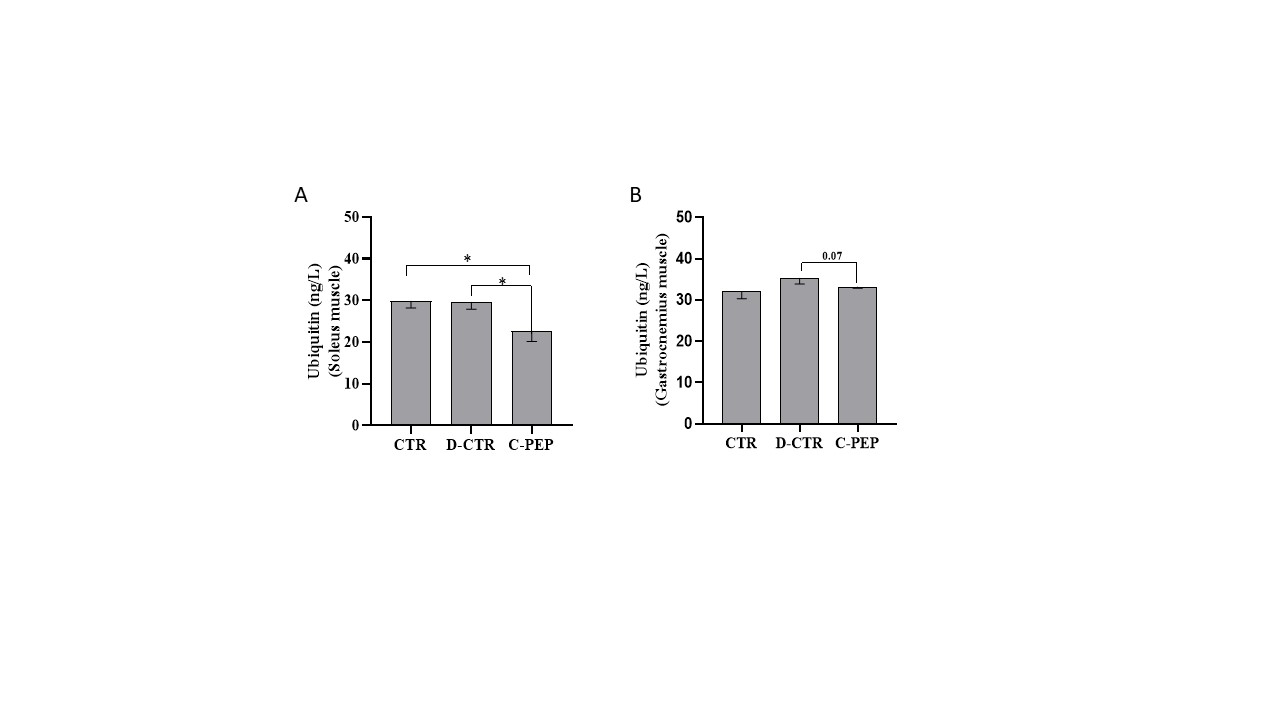


Suppl.Fig.1: Levels of ubiquitin in SO were lower in the C-peptide group than diabetic control rats. (A) Ubiquitin levels of SO (B) and GC were measured by Elisa Kit (n=3 (CTR), n=2(D-CTR) and n=3 (C-PEP). Data are represented as the means ± SD. Statistical analysis: Student’s t-test * p < 0.05


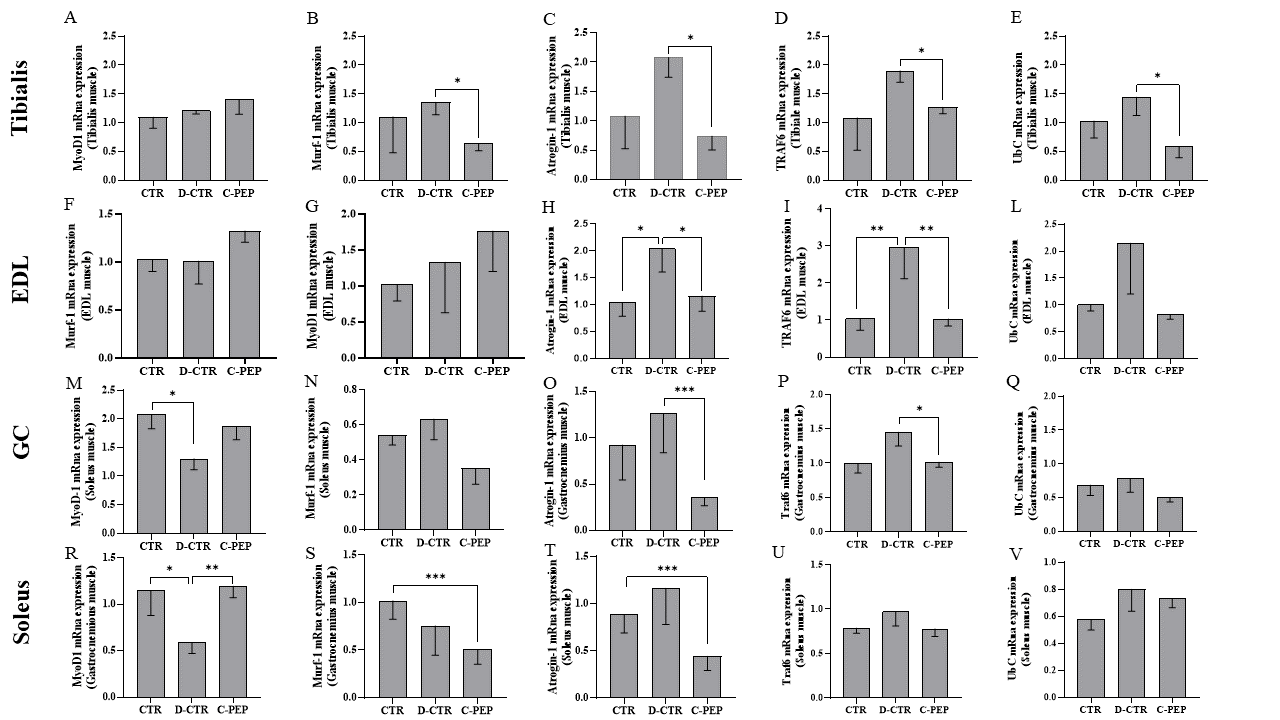


Suppl. Fig. 2. C-peptide increases MyoD-1 mRna espression, in GC (M), downregulates Murf-1 (B), UbC (E) Atrogin-1 and Traf6 mRna espression in TA (C and D), EDL (H and I) and GC (O and P) muscles. Data are represented as the means ± SD (CTR (n.3 rats), D-CTR (n.2 rats), C-PEP (n.3 rats)). Statistical analysis: Student’s t-test * p < 0.05 **p<0.01 and ***p<0.001.


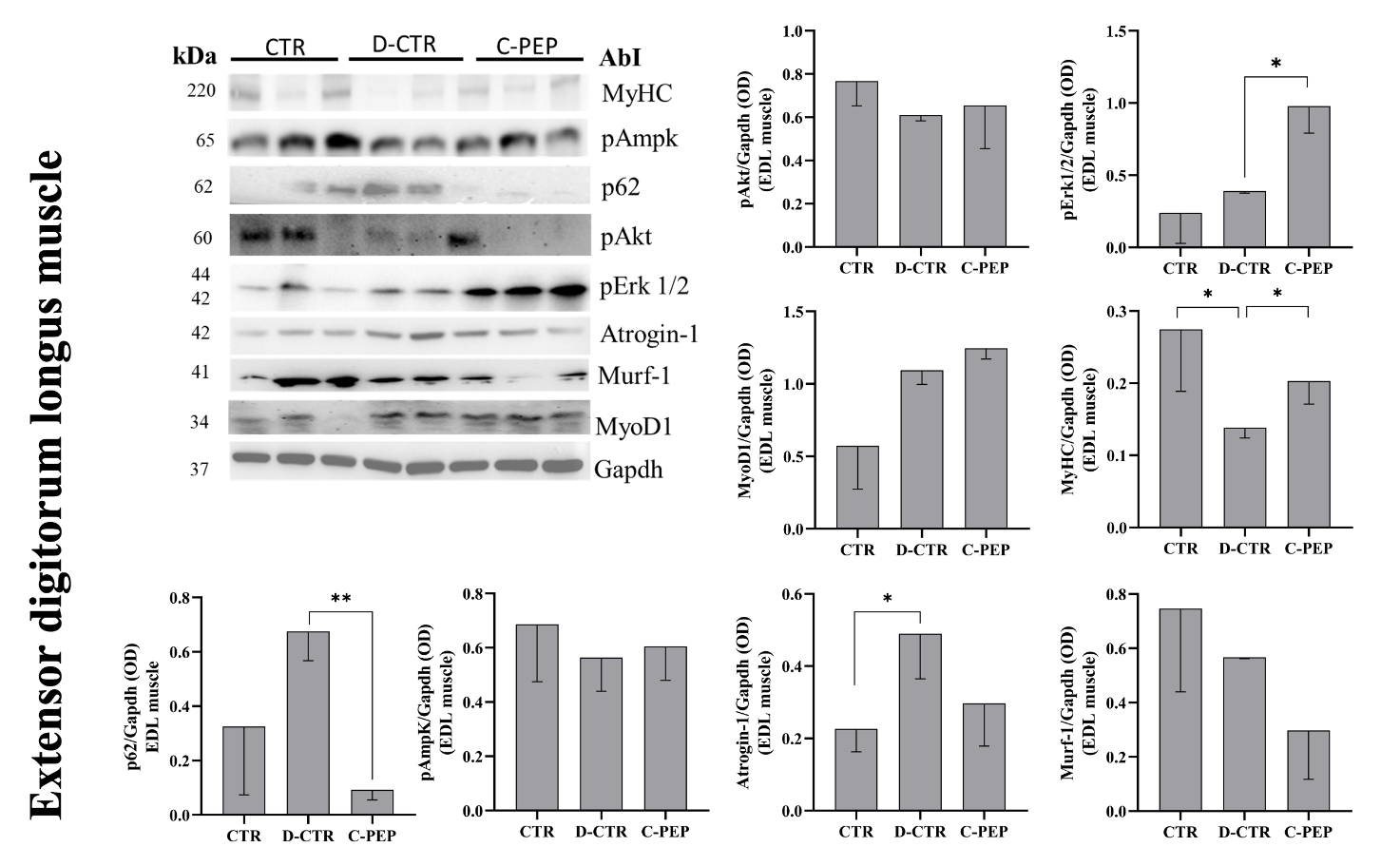


Suppl. Fig. 3. C-peptide increases pErk1/2 and MyHC and decreases p62 protein levels on EDL muscle. All proteins expression levels were analyzed by Western blotting with specific antibodies. Data are represented as mean ± SD of three independent experiments, in a subgroup of rats (n=3 (CTR), n=2 (D-CTR) and n=3 (C-PEP)). Statistical analysis: Student’s t-test * p < 0.05 ** p<0.01


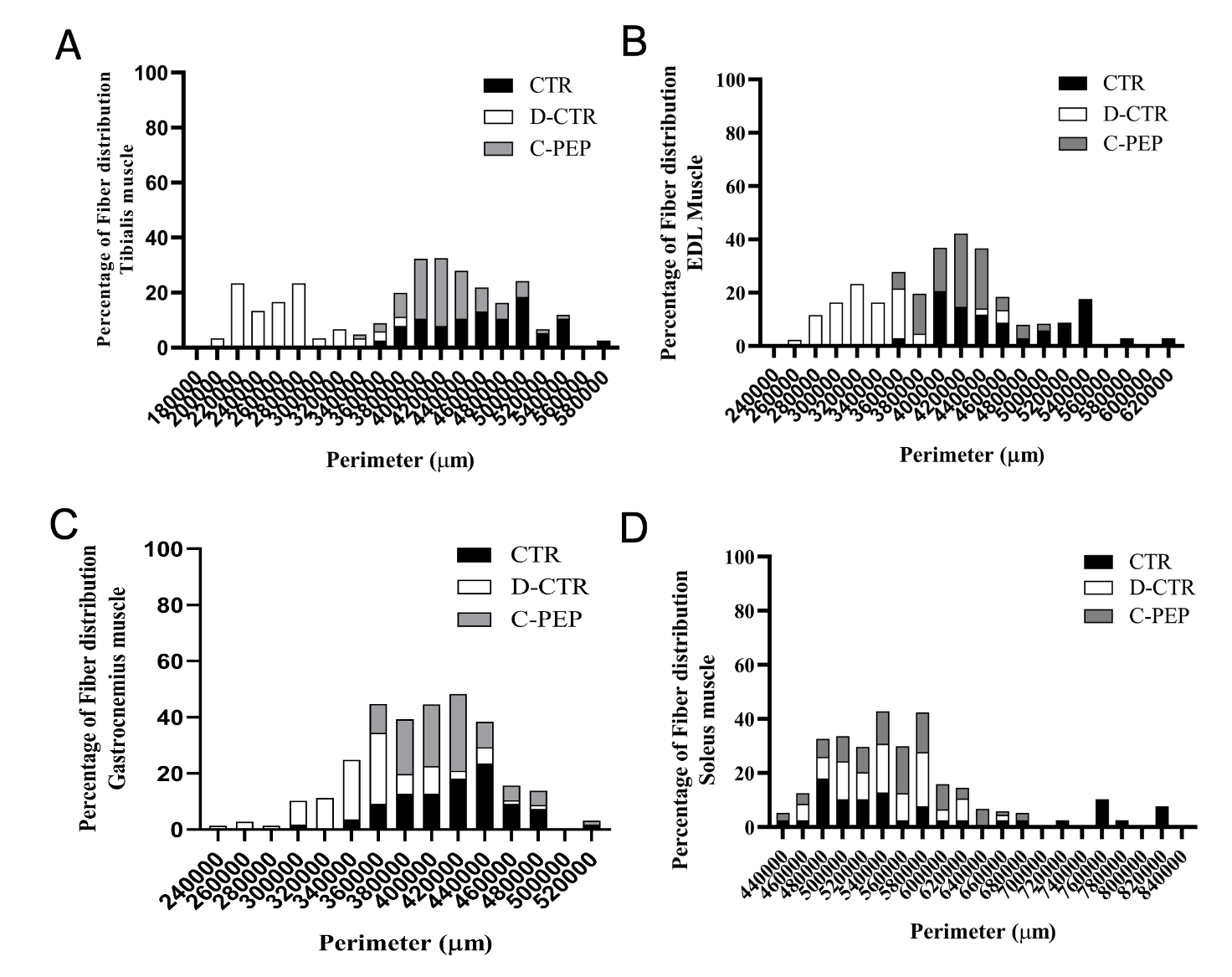


Suppl. Fig. 4. Percentage distribution of fibers’ perimeters in GC, EDL, TA and SO. Data are represented as the means ± SD (n=6 (CTR), n=6 (D-CTR) and n=8 (C-PEP).
